# Supplementary material for: Ethnic inequalities in patient satisfaction with primary health care in England: Evidence from recent General Practitioner Patient Surveys (GPPS)
Source: PLoS One. 2022 Dec 21;17(12):e0270775. doi: 10.1371/journal.pone.0270775 (PMC9770381; doi:10.1371/journal.pone.0270775)
Supplement: S1 File — (DOCX) [file pone.0270775.s001.docx]

#### Annex (i): GPPS variables extracted and considered in the analysis

| **Variable** | **Description** |
| --- | --- |
| Q3_12pct | Ease of getting through to someone at GP practice on the phone |
| Q4_12pct | Helpfulness of receptionists at GP practice - % Summary result |
| Q73_12pct | Ease of using your GP practice's website to look for information or access services |
| Q25_12pct | Satisfaction with general practice appointment times |
| Q8_14pct | Have a preferred GP - % Summary result |
| Q9_12pct | Frequency of seeing preferred GP |
| Q100_4pct | Actions taken before trying to get an appointment |
| Q103_1234pct | Whether offered a choice of appointment |
| Q104_12pct | Satisfaction with type of appointment offered |
| Q18_12pct | Overall experience of making an appointment |
| Q86a_12pct | Last time you had a general practice appointment, how good was the healthcare professional at: Giving you enough time |
| Q86b_12pct | Last time you had a general practice appointment, how good was the healthcare professional at: Listening to you |
| Q86e_12pct | Last time you had a general practice appointment, how good was the healthcare professional at: Treating you with care and concern |
| Q88_12pct | During last general practice appointment, involved as much as wanted to be in decisions about care and treatment |
| Q89_12pct | During last general practice appointment, had confidence and trust in healthcare professional |
| Q90_12pct | Needs met at last general practice appointment |
| Q28_12pct | Overall experience of GP practice |
| Q30_recoded_1pct | Long-term health condition |
| Q94_12pct | Confidence can manage any issues arising from your condition (or conditions) |
| Q32_12pct | Last 12 months, had enough support from local services/organisations to help manage long |
| Q97_1pct | Agreed a plan with a healthcare professional from your GP practice to manage your condition (or conditions) |
| Q70_12pct | Overall experience of NHS services when GP practice was closed |
| Q110_12pct | In last 12 months, have you or household member shielded at home due to being vulnerable to COVID |
| Q111_12345pct | Avoided making a general practice appointment in last 12 months |
| Q48_Merged_1pct | Age - % Under 16 |
| Q48_Merged_2pct | Age - % 16 to 24 |
| Q48_Merged_3pct | Age - % 25 to 34 |
| Q48_Merged_4pct | Age - % 35 to 44 |
| Q48_Merged_5pct | Age - % 45 to 54 |
| Q48_Merged_6pct | Age - % 55 to 64 |
| Q48_Merged_7pct | Age - % 65 to 74 |
| Q48_Merged_8pct | Age - % 75 to 84 |
| Q48_Merged_9pct | Age - % 85 or over |
| Q50_1pct | Working status - % In full-time paid work (30 hours or more each week) |
| Q50_2pct | Working status - % In part-time paid work (under 30 hours each week) |
| Q50_3pct | Working status - % In full-time education at school, college or university |
| Q50_4pct | Working status - % Unemployed |
| Q50_5pct | Working status - % Permanently sick or disabled |
| Q50_6pct | Working status - % Fully retired from work |
| Q50_7pct | Working status - % Looking after the family or home |
| Q50_8pct | Working status - % Doing something else |
| Q57_1pct | Sexual orientation - % Heterosexual or straight |
| Q57_234pct | Sexual orientation - % Summary result - LGB+ (Combined 'gay or lesbian', 'bisexual' and 'other' responses, to be used with total base) |
| Q58_1pct | Religion - % No religion |
| Q58_2pct | Religion - % Buddhist |
| Q58_3pct | Religion - % Christian |
| Q58_4pct | Religion - % Hindu |
| Q58_5pct | Religion - % Jewish |
| Q58_6pct | Religion - % Muslim |
| Q58_7pct | Religion - % Sikh |
| Q58_8pct | Religion - % Other |
| Q58_9pct | Religion - % I would prefer not to say |
| Q49_1pct | White English/Welsh/Scottish/Northern Irish/British |
| Q49_2pct | White Irish |
| Q49_3pct | White Gypsy or Irish Traveller |
| Q49_4pct | Ethnic group - % White - Any other White background |
| Q49_5pct | Ethnic group - % Mixed - White and Black Caribbean |
| Q49_6pct | Ethnic group - % Mixed - White and Black African |
| Q49_7pct | Ethnic group - % Mixed - White and Asian |
| Q49_8pct | Ethnic group - % Mixed - Any other Mixed/Multiple Ethnic background |
| Q49_9pct | Ethnic group - % Asian/Asian British - Indian |
| Q49_10pct | Ethnic group - % Asian/Asian British - Pakistani |
| Q49_11pct | Ethnic group - % Asian/Asian British - Bangladeshi |
| Q49_12pct | Ethnic group - % Asian/Asian British - Chinese |
| Q49_13pct | Ethnic group - % Asian/Asian British - Any other Asian background |
| Q49_15pct | Ethnic group - % Black/African/Caribbean/Black British - Caribbean |
| Q49_14pct | Ethnic group - % Black/African/Caribbean/Black British - African |
| Q49_16pct | Ethnic group - % Black/African/Caribbean/Black British - Any other Black/African/Caribbean background |
| Q49_17pct | Ethnic group - % Other – Arab |
| Q49_18pct | Ethnic group - % Other - Any other ethnic group |
| ***Derived ethnicity*** |  |
| White | Q49_1pct + Q49_2pct + Q49_3pct + Q49_4pct |
| Mixed | Q49_5pct + Q49_6pct + Q49_7pct + Q49_8pct. |
| Indian | Q49_9pct. |
| Pakistani | Q49_10pct |
| Bangladeshi | Q49_11pct |
| Other Asian | Q49_12pct + Q49_13pct. |
| All Asian | Q49_9pct + Q49_10pct + Q49_11pct + Q49_12pct + Q49_13pct. |
| Black | Q49_14pct + Q49_15pct + Q49_16pct. |
| Arab and other | Q49_17pct + Q49_18pct. |
| All minority | Q49_5pct+ Q49_6pct +Q49_7pct+Q49_8pct+ Q49_9pct +Q49_10pct+ Q49_11pct +Q49_12pct+ Q49_13pct+ Q49_14pct +Q49_15pct +Q49_16pct+ Q49_17pct+ Q49_18pct. |
