# Supplementary material for: Ethnic inequalities in patient satisfaction with primary health care in England: Evidence from recent General Practitioner Patient Surveys (GPPS)
Source: PLoS One. 2022 Dec 21;17(12):e0270775. doi: 10.1371/journal.pone.0270775 (PMC9770381; doi:10.1371/journal.pone.0270775)
Supplement: S2 File — (DOCX) [file pone.0270775.s002.docx]

#### Annex (ii): Bivariate correlation analysis of patient satisfaction by ethnicity (England - 2019, 2020 & 2021)

| **Ethnicity** | | **Overall satisfaction (% ‘v.good’ or ‘fairly good’ experience)** | | | |
| --- | --- | --- | --- | --- | --- |
|  |  | **2019** | **2020** | **2021** | **All** |
| White | Pearson Correlation | .357^**^ | .342^**^ | .352^**^ | .348^**^ |
|  | Sig. (2-tailed) | <.001 | <.001 | <.001 | <.001 |
|  | N | 6303 | 6249 | 5790 | 18342 |
| Mixed | Pearson Correlation | -.109^**^ | -.109^**^ | -.122^**^ | -.113^**^ |
|  | Sig. (2-tailed) | <.001 | <.001 | <.001 | <.001 |
|  | N | 6527 | 6377 | 5923 | 18827 |
| Indian | Pearson Correlation | -.213^**^ | -.212^**^ | -.212^**^ | -.211^**^ |
|  | Sig. (2-tailed) | <.001 | <.001 | <.001 | <.001 |
|  | N | 6759 | 6595 | 6337 | 19691 |
| Pakistani | Pearson Correlation | -.259^**^ | -.246^**^ | -.261^**^ | -.255^**^ |
|  | Sig. (2-tailed) | <.001 | <.001 | <.001 | <.001 |
|  | N | 6845 | 6667 | 6465 | 19977 |
| Bangladeshi | Pearson Correlation | -.211^**^ | -.195^**^ | -.194^**^ | -.199^**^ |
|  | Sig. (2-tailed) | <.001 | <.001 | <.001 | <.001 |
|  | N | 6879 | 6692 | 6510 | 20081 |
| Other Asian | Pearson Correlation | -.156^**^ | -.147^**^ | -.153^**^ | -.151^**^ |
|  | Sig. (2-tailed) | <.001 | <.001 | <.001 | <.001 |
|  | N | 6621 | 6450 | 6073 | 19144 |
| All Asian | Pearson Correlation | -.347^**^ | -.324^**^ | -.333^**^ | -.334^**^ |
|  | Sig. (2-tailed) | <.001 | <.001 | <.001 | <.001 |
|  | N | 6356 | 6228 | 5671 | 18255 |
| Black | Pearson Correlation | -.202^**^ | -.209^**^ | -.228^**^ | -.211^**^ |
|  | Sig. (2-tailed) | <.001 | <.001 | <.001 | <.001 |
|  | N | 6654 | 6475 | 6072 | 19201 |
| Arab or other | Pearson Correlation | -.204^**^ | -.193^**^ | -.191^**^ | -.196^**^ |
|  | Sig. (2-tailed) | <.001 | <.001 | <.001 | <.001 |
|  | N | 6717 | 6521 | 6220 | 19458 |
| All minority ethnic | Pearson Correlation | -.356^**^ | -.337^**^ | -.351^**^ | -.347^**^ |
|  | Sig. (2-tailed) | <.001 | <.001 | <.001 | <.001 |
|  | N | 5665 | 5566 | 4531 | 15762 |

** - p<0.001
