# Supplementary material for: Ethnic inequalities in patient satisfaction with primary health care in England: Evidence from recent General Practitioner Patient Surveys (GPPS)
Source: PLoS One. 2022 Dec 21;17(12):e0270775. doi: 10.1371/journal.pone.0270775 (PMC9770381; doi:10.1371/journal.pone.0270775)
Supplement: S3 File — (DOCX) [file pone.0270775.s003.docx]

#### Annex (iii): Stepwise multilevel linear regression results of predictors of patient satisfaction (England 2021)

| **Factor** | **Model 0** | **Model 1** | **Model2** | **Model3a** | **Model3b** | **Model3c** | **Model3d** | **Model 3** |
| --- | --- | --- | --- | --- | --- | --- | --- | --- |
| Constant | 0.84 | 0.86 | 0.946 | 0.493 | 0.497 | -0.082 | -0.134 | -0.359 |
| Minority ethic (%) |  | -0.15 ** | -0.17** | -0.10** | -0.09** | -0.02** | -0.01 | 0.00 |
| Long-term Health condition (Q30_recoded_1pct) |  |  | -0.15** | -0.08** | -0.05** | -0.02* | -0.01 | 0.00 |
| Ease of using website (Q73_12pct) |  |  |  | 0.529** | 0.39** | 0.23** | 0.23** | 0.22** |
| Always seen preferred GP (Q9_12pct) |  |  |  |  | 0.18** | 0.12** | 0.12** | 0.12** |
| Care and concern (Q86e_12pct) |  |  |  |  |  | 0.79** | 0.78** | 0.61** |
| Confidence of managing condition (Q94_12pct) |  |  |  |  |  |  | 0.07** | 0.05** |
| Trust in healthcare professional (Q89_12pct |  |  |  |  |  |  |  | 0.23** |
| Involved in decisions about treatment (Q88_12pct) |  |  |  |  |  |  |  | 0.18** |
| ***Random effects*** |  |  |  |  |  |  |  |  |
| CCG variance | 0.00098** | 0.00071** | 0.00067** | 0.00026** | 0.00019** | 0.00004** | 0.00004** | 0.00004** |
| ICC | 0.111 | 0.089 | 0.085 | 0.056 | 0.051 | 0.018 | 0.018 | 0.019 |

* -p<0.05; **p<0.01;
