# Supplementary material for: Ethnic inequalities in patient satisfaction with primary health care in England: Evidence from recent General Practitioner Patient Surveys (GPPS)
Source: PLoS One. 2022 Dec 21;17(12):e0270775. doi: 10.1371/journal.pone.0270775 (PMC9770381; doi:10.1371/journal.pone.0270775)
Supplement: Attachment — Submitted filename: Response to reviewers (Oct 2022).docx [file pone.0270775.s004.docx]

**Response to reviewers**

We very much appreciate the invaluable comments from the reviewers which we believe have helped improve the quality of the paper. Our response to each of the specific comments raised by the reviewers is provided in the table below.

| **Comment** | **Response** |
| --- | --- |
| ***Reviewer #1*** | |
| This was a well written manuscript, there were two statements that the researchers made without any reference. Line 60 - Researchers mentioned interventions that were previously done, it would be interesting to know what these interventions were in order to compare with results of the current study and see if any of those interventions would be applicable. | Thank you! The introduction (including sentence making reference to interventions) has been revised to make it more concise, in response to comments by the second reviewer. |
| Line 111-112 the statement made has no reference ; however I saw in the results sections in the researchers did reference teh statement. | Thank You! The statement has been re-framed as suggested by the second reviewer (see #4 below). |
| ***Reviewer #2*** | |
| As the surveys are yearly, 3 years is not long enough to examine a trend, although this study provides recent evidence that can be interpreted in the context of previous work. Removing references to the trend element would help to focus on the rest of the analysis. | Agreed. The reference to trend (i.e second objective) has been removed to help refine focus of the paper. |
| A key improvement would be to give more detail about the ethnic groups used in the analysis. The surveys included used different ethnic groups, and the list provided in the Annex does not include all of the White ethnic groups. Describing in the Methods or providing a key in the Annex showing which groups were included in the groups analysed would be helpful. Some of the White groups would be described as ethnic minority groups, and have been shown to have different responses to the GPPS than the White British group (e.g. Burt et al Br J Gen Pract 2016;66(642):e47-52) although the numbers would probably have been too small to analyse. Were they included in the broad White group or excluded? Does the “Other Asian” group analysed include the Chinese group or is it just the “Any other Asian background” group? The Asian census category in England and Wales changed to include the Chinese group in 2011, and it is helpful to be clear about the categories used. The terms “Southeast Asians”, “South Asians” and “Asians” are all included in the manuscript – it would be useful to describe the differences between these groups, or use (and define) a single term if they are referring to the same group. | Annex (i) has been revised to include the derived ethnicity variables, showing which specific ethnic groups in the original data were used to derive specific ethnic categories. It was not possible to separate specific White (or other) ethnic categories since numbers were too small to enable meaningful analysis . |
| ***Minor comments:*** |  |
| 1. The Introduction is very long, and several sections could be omitted as they are addressed in the Discussion. Indeed, the whole manuscript would benefit from some editing to make it easier to read and checking grammatical and spelling errors. | The introduction has been revised and made more concise. Also, the whole manuscript has been further edited to correct for grammatical and spelling errors. |
| 2.There are several statements in the Introduction which need to be supported by references (e.g. lines 56-59). | Done – factual statements which are not common knowledge (e.g lines 56-59) now supported with relevant references. |
| 3.Line 66. It is not obvious how “genetic factors” or “tougher living conditions” could directly influence patient satisfaction. If the implication is that these factors result in poorer health, which is associated with lower satisfaction scores, this should be made clearer. | Agreed - The statement has been revised to clarify this. |
| 4. There should be a balance in the language to try and make statements more neutral when there is no evidence of the causes or intention behind study results. For example, lines 111-112 “Therefore, it is plausible that ethnic minorities dealing with a variety of health issues may exhibit their frustrations subconsciously on their patient satisfaction scores […]” could easily be framed as “[…] with a variety of health issues may not be having their complex needs met and report this in their patient satisfaction scores […]”. | Thank you! The sentence has been re-framed as suggested. |
| 5. England and the UK are often used interchangeably – all of the data analysed are from England, and national representation may not mean it is possible to generalise to the UK (line 218). | This has been corrected and reference made to England as appropriate. |
| 6. Methods. How were GP practices that were in more than one survey treated? What was the overlap? | All analyses presented either examine each of the surveys separately (e.g. descriptive analysis), or are based on the most recent survey at the time of analysis (i.e 2021) only (e.g multilevel analysis), so GP practices appearing in more than one survey is not an issue. |
| 7. Line 225. Remove reference to “most recent” as the 2022 survey is now available. | Done – reference made to ‘recent’ rather than ‘most recent’ surveys. |
| 8. The Data Limitations section should be moved to the Discussion, possibly as part of a Strengths and Limitations section. | Done – the Data Limitations section has been moved to the discussion section on ‘Strengths and limitations (4.3). |
| 9. Table 2. This should either be labelled as “Proportion” or the numbers changed to percentages. Please update the ethnicity categories labels to remove “_”. It is also unclear what the N column refers to, please add a description. | Done. Table 2 labelled as proportion, ethnicity updated to remove’_’, and N clarified as GPs. |
| 10. All Tables and Figures should include the geography and years studied in the title. | Done. All tables now include geography (i.e England) and year in title |
| 11. The (Field 2009) references should be cited fully. | Done. Details of Field (2009) now provided in reference list. |
| 12. I’d suggest removing Table 3 and reporting the mean and 95% confidence intervals in the text. | Done. Table 3 removed, and means and confidence intervals reported in text. |
| 13. Line 378. Could either report that (r<\|0.4\|) or (-0.414. Line 379. Suggest rephrasing “most negative correlation” to “strongest negative correlation”. | Done – revised (r<0.4) to (r<\|0.4\|), and replaced ‘most negative correlation’ with ‘strongest negative correlation’. |
| 15. Table 4. Suggest moving the table to the Appendix. Please update the ethnicity categories labels to remove “_” and describe “minority” better. This could also be simplified, as all the p-values are <0.001 (not 0.000) and the ** label is not needed (or defined). | Done – Table 4 edited as suggested and moved to Annex (ii) |
| 16. Figure 1. Please add a y-axis title and change the x-axis title to clarify that it’s showing the % of ethnic minority respondents in each GP practice. | Figure 1 revised to make x & y axes labels clearer |
| 17. Figures 2 and 3. Please give the x and y-axes more descriptive titles. It might be easier to read if the scales were converted to percentages. | More descriptive titles for x and y axes provided in Figures 2 & 3 |
| 18. Lines 562 and 646. The ‘Care and concern’ does not necessarily act as a proxy for “cultural differences and communication barriers”. Is there any evidence that these factors influence respondents’ ratings for the ‘care and concern’ question? Similarly (line 635), Ease of using GP website *may* relate to issues of access and language barriers. | We do agree that ‘care and concern’ does not necessarily act as a proxy for “cultural differences and communication barriers”. The sentence has been revised to “…that ethnic minorities would be more likely to negatively rate the ‘Care and concern’ they received partly due to cultural differences and communication barriers…”, to reflect the possible contribution of cultural differences and communication barriers to the negative rating of ‘care and concern’. Also, the sentence on ease of using GP website has been revised to include issues of access and language barriers. |
| 19. There should be some discussion in the (Strengths and) Limitations about the difference between individual level ethnicity and proportion of respondents from ethnic groups. | A paragraph (and relevant reference [36]) has been added at the end of ‘strengths and limitations’ section to address this. |
